# Fishbone-Induced Appendicitis: A Case Report

**DOI:** 10.7759/cureus.15003

**Published:** 2021-05-13

**Authors:** Marouane Harhar, Rachid Jabi, Tijani El Harroudi, Mohammed Bouziane

**Affiliations:** 1 Surgical Oncology, Mohammed VI University Hospital, Regional Oncology Center, Oujda, MAR; 2 Visceral Surgery, Mohamed VI University Hospital, Oujda, MAR; 3 General Surgery , Surgical Oncology, Mohammed VI University Hospital Centre, Oujda, MAR

**Keywords:** fishbone, acute appendicitis

## Abstract

Fishbone ingestion is quite common. Most of the time, patients are asymptomatic and the fish bone exits the gastrointestinal tract spontaneously. However, in some rare cases, it can drop in the appendix and induce appendicitis or even appendicitis with perforation. Herein, we report the unusual case of an 18-year-old woman, who presented with acute right lower abdominal pain. Computed tomography suggested the presence of acute appendicitis with a linear foreign body of 3 cm in length. The patient underwent an open appendectomy and removal of the fish bone without stigmata of perforation. The postoperative course was uneventful.

## Introduction

Acute appendicitis is one of the major causes of acute abdominal pain in the lower right quadrant. Rarely, appendicitis can be induced by ingested foreign bodies that drop in the appendix [1]. The fish bone is one of the most ingested foreign objects that have been rarely reported in the literature as a cause for acute appendicitis [2]. They are majorly responsible for gastrointestinal tract perforation [3]. Here, we describe the case of a fishbone-induced nonperforated appendicitis in an 18-year-old patient.

## Case presentation

An 18-year-old female patient presented to the emergency room with acute abdominal pain in the lower right quadrant associated with nausea and vomiting. The physical examination showed local tenderness in the right lower quadrant.

**Figure 1:**
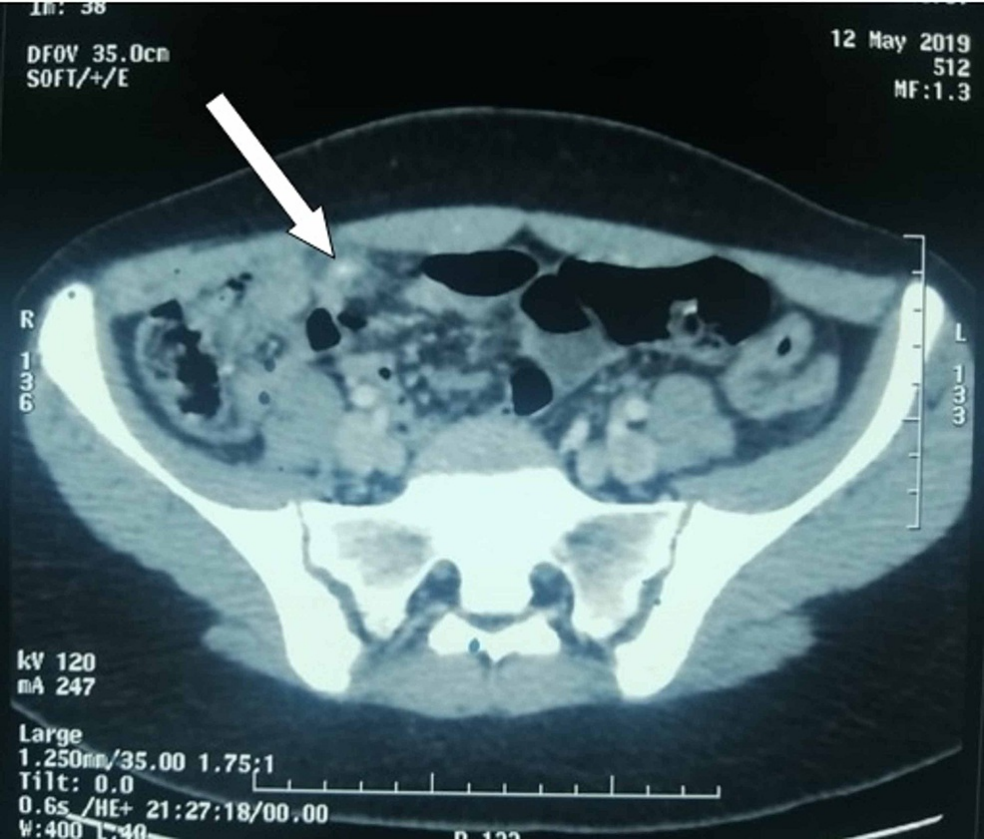
Axial contrast-enhanced CT showing of the abdomen showing dilated appendix associated with appendiceal wall thickening in favor of acute appendicitis and the presence of a linear spontaneously hyperdense image of 3 cm in length (white arrow)

Laboratory tests showed elevation C-reactive protein with normal white cell count. The Alvarado score was 6/10. Abdominal ultrasound showed a dilated appendix associated with appendiceal wall thickening measuring 6.5 mm alongside the presence of a linear foreign body. Thus, a contrast-enhanced CT scan was performed and it revealed evidence of acute appendicitis, as well as the presence of a spontaneously hyperdense, linear image measuring 3 cm in length (Figure 1). In this regard, we conducted a thorough investigation with the patient; however, she denied any kind of object ingestion over the two precedent weeks.

**Figure 2:**
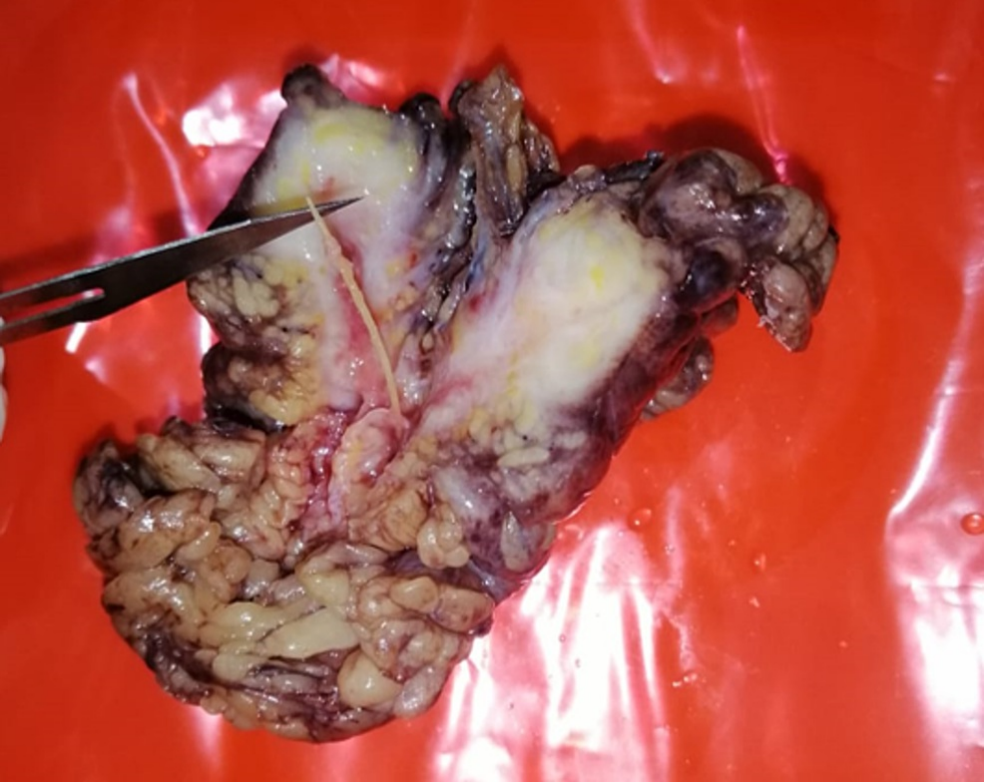
Specimen showing the fish bone surrounded by the omental granuloma

The patient underwent open surgery that showed an inflamed appendix and the presence of an omental granuloma with the fish bone inside it. An appendectomy was performed and the omental granuloma was removed alongside the fish bone (Figure 2).

No stigmata of gastrointestinal perforation were found. The patient made an uneventful recovery and was discharged from the hospital on postoperative day 2.

## Discussion

The fish bone is one of the most ingested foreign objects. Mostly, fishbone ingestion can be asymptomatic; however, it can get impacted in any part of the digestive tract and cause serious complications, such as perforation, abscess, and tract obstruction [4]. Terminal ileum and duodenal C-loop are the most prevalent sites of gastrointestinal tract perforation due to ingested sharp foreign bodies [5].

The incidence of fishbone-induced appendicitis is still unknown; nonetheless, foreign objects in the appendix have been reported in 0.005% to 0.113% of cases [6].

When the foreign object drops in the appendix, its poor peristaltic movement is unable to expel the object back to the cecum, leading progressively to inflammation with a high risk of perforation [2]. In our case, the fish bone induced acute appendicitis without evident perforation of the gastrointestinal tract.

The diagnosis of acute appendicitis can be easily made in 80% of cases based on symptoms and physical examination, but in the other 20% of cases the diagnosis may be difficult and different means of imaging will be needed [7].

Laboratory assessments show classically elevated inflammatory markers. Abdominal radiography is not reliable in finding the fish bone. The plain radiography has a sensitivity of only 32% of detecting fish bones according to several studies [8,9]. CT scan remains the means of choice in finding ingested foreign objects. The fishbone perforation appears as a linear calcified object surrounded by inflammation [2,10]. This imaging modality has 100% sensitivity and 97.8% specificity rates [10].

Surgical treatment is the gold standard in the case of fishbone-induced appendicitis [6]. In our case, the patient underwent open appendectomy and removal of the fish bone alongside the omental mass surrounding it; however, the abdominal exploration did not find any evident perforation. The postoperative course was uneventful.

Generally, patients do not recall ingesting fish bones. Due to their small size, they tend to pass through the gastrointestinal tract without incidence or may cause nonspecific symptoms, which decreases the chances of preoperative diagnosis of fishbone-induced appendicitis. A meticulous investigation should always be done with the patient whenever a foreign body is suspected on a CT scan.

## Conclusions

This paper illustrates an unusual case of fishbone-induced appendicitis without any stigmata of perforation. The fish bone is one of the most ingested foreign bodies. However, in some rare cases, it can drop in the appendix and induce appendicitis or even appendicitis perforation. CT scan remains the means of choice in finding ingested foreign objects. Surgical treatment is the gold standard in case of fishbone-induced appendicitis.
